# Exploring Platelet Indices as Predictors of Nephropathy Severity in Type 2 Diabetes Mellitus: A Hospital-Based Cross-Sectional Analysis

**DOI:** 10.7759/cureus.71796

**Published:** 2024-10-18

**Authors:** Piyali Sengupta, Aparajita Priyadarshini, Pradip Kumar Behera, Krishna Padarabinda Tripathy

**Affiliations:** 1 General Medicine, Kalinga Institute of Medical Sciences, Bhubaneswar, IND; 2 Physiology, Kalinga Institute of Medical Sciences, Bhubaneswar, IND

**Keywords:** mean platelet volume (mpv), microvascular complications, plateletcrit, platelet distribution width, platelet-large cell ratio

## Abstract

Background

Increased platelet activity in type 2 diabetes mellitus (T2DM) plays a key role in the development of vascular complications. Platelet indices such as mean platelet volume (MPV), platelet distribution width (PDW), platelet large cell ratio (PLCR), and plateletcrit (PCT) reflect both the functional and morphological status of platelets. These indices are markers of inflammation and metabolic dysregulation, which are pivotal in diabetic vasculopathy. If platelet indices correlate with nephropathy severity, they could be used as cost-effective, accessible markers for assessing disease progression in T2DM.

Materials and methods

This cross-sectional study was conducted at Kalinga Institute of Medical Sciences (KIMS), Bhubaneswar, India, from September 2019 to August 2021. A total of 203 patients with T2DM and nephropathy were included, diagnosed per American Diabetes Association 2017 criteria and staged using Kidney Disease: Improving Global Outcomes (KDIGO) guidelines. Nephropathy was assessed using the albumin-to-creatinine ratio, and the glomerular filtration rate (GFR) was calculated using the Modification of Diet in Renal Disease (MDRD) formula. Venous blood samples were collected to measure platelet indices using the SYSMEX XN-1000 automated analyzer (Sysmex Corporation, Kobe, Japan). Statistical analysis was conducted using Statistical Package for the Social Sciences (IBM SPSS Statistics for Windows, IBM Corp., Version 24.0, Armonk, NY), with a comparison of MPV, PDW, PCT, and PLCR across nephropathy stages performed via the Kruskal-Wallis test. Pairwise comparisons were made using the Mann-Whitney test, with a significance level set at p < 0.05.

Results

The study population had a mean age of 61.7 ± 12.0 years, with 62.1% over 60 years and a male-to-female ratio of 1.5:1. The average diabetes duration was 8.0 ± 5.2 years. The mean platelet count was 236.4 ± 112.6, and the mean values for MPV, PDW, PCT, and PLCR were 11.4 ± 1.7, 15.2 ± 3.8, 0.28 ± 0.11, and 38.9 ± 11.8, respectively. Most patients (63.1%) were in the early stages of nephropathy (stages 1-3). Significant differences in platelet indices were observed across nephropathy stages, with Kruskal-Wallis p-values of 0.027, 0.009, 0.001, and 0.007 for MPV, PDW, PCT, and PLCR, respectively. Pairwise comparisons showed that platelet indices were significantly elevated in advanced nephropathy stages (stages 4 and 5) compared to early stages (stages 1 and 2) with p < 0.05.

Conclusion

There is a significant correlation between platelet indices and the severity of nephropathy in T2DM patients. MPV, PDW, PCT, and PLCR all increase in advanced stages of nephropathy, suggesting these indices can be used as surrogate markers for assessing disease progression. Their ease of measurement and cost-effectiveness make them valuable tools in monitoring diabetic nephropathy, offering a simpler and more cost-effective alternative for monitoring disease progression.

## Introduction

Diabetes mellitus (DM) is a chronic metabolic disorder characterized by persistent hyperglycemia due to either insulin deficiency or insulin resistance, resulting in vascular complications across various organs [1]. Type 2 DM accounts for 90% of diabetes cases worldwide [2]. DM has become a global health challenge, with the number of cases rising to a staggering 463 million (8.8% of adults) worldwide by 2019, and it is expected to rise to 700 million (10.9%) by 2045. It is estimated that middle- and low-income countries will contribute around 80% of the global diabetes burden [3]. The Indian population has a genetic predisposition to type 2 DM, and the prevalence in the Indian subcontinent is rising alarmingly. In 2019, 77 million people in India had diabetes, with projections indicating that this will rise to over 134 million by 2045 [4]. The majority of morbidity and mortality associated with DM is due to vascular complications arising from metabolic dysregulation caused by chronic hyperglycemia, leading to significant costs and decreased quality of life for patients and their relatives, while also placing an increasing burden on healthcare systems.

Sustained hyperglycemia in DM leads to endothelial dysfunction and chronic inflammation, which results in microvascular complications in the nerves, kidneys, and retina, as well as atherosclerotic macrovascular changes in larger arteries, including those in the brain, heart, and peripheral arteries [5]. Though macrovascular complications are responsible for the majority of morbidity and mortality in DM, microvascular complications often occur earlier. Diabetic nephropathy, also referred to as diabetic kidney disease, is a major contributor to morbidity in diabetic individuals and is the leading cause of end-stage renal disease (ESRD) worldwide [6]. Diabetic nephropathy is characterized by progressive kidney damage, reflected by increasing albuminuria and a gradual decline in glomerular filtration rate (GFR), ultimately leading to ESRD [7].

To reduce the burden of morbidity and mortality, screening diabetic patients for relevant complications is an essential aspect of diabetes care today. Early detection allows for focused preventive treatment and specific management, which can delay the progression of these complications.

Although elevated albuminuria is a standard method for screening and assessing the severity of diabetic nephropathy, urinary albumin excretion can be influenced by factors unrelated to renal pathology, such as severe exercise within 24 hours, urinary tract infections, menstruation, marked hyperglycemia, or heart failure [8]. Therefore, current research is focused on developing new methods for the early detection of diabetic kidney disease, including the identification of new biomarkers in the blood and urine. In diabetic individuals, altered platelet morphology and function have been linked to vascular pathology [9,10].

Platelets play a significant role in the development and progression of thrombosis and atherosclerotic lesions [11]. Given the low cost of peripheral blood smear or platelet index analysis and the role of platelets in inflammation and endothelial dysfunction, in recent years many researchers have explored the association between platelet morphological indices and microvascular complications in diabetes [12-14]. Platelet indices like mean platelet volume (MPV), platelet distribution width (PDW), platelet large cell ratio (PLCR), and plateletcrit (PCT) are being studied for their association with glycemic control and disease duration. Taderegew MM et al. observed significantly higher MPV, PDW, and PLCR values in diabetics with microvascular complications compared to those without microvascular complications [15]. Similarly, Subramanian S et al. and Dubey I et al. reported similar observations in their studies [16,17]. In a meta-analysis, Liu J et al. found MPV and PDW to be significantly associated with diabetic retinopathy and nephropathy [18]. However, there is a scarcity of literature on whether platelet indices correlate with different stages of diabetic kidney disease [19,20]. In this study, we aim to explore the correlation between platelet indices and stages of diabetic nephropathy, with the goal of establishing platelet indices as potential markers for predicting the severity of diabetic kidney disease in individuals with type 2 DM.

## Materials and methods

The study was carried out in the Department of Internal Medicine, Kalinga Institute of Medical Sciences, Bhubaneswar, India, as a hospital-based cross-sectional study from September 2019 to August 2021. The study protocol was approved by the Institutional Ethics Committee (ref no.: KIMS/KIIT/IEC125/2019).

Sample size

The required sample size was calculated using the formula:

\[n = \frac{z_{(1-\alpha/2)}^2 \cdot P \cdot (1 - P)}{d^2}\]

where n represents the minimum sample size,

​​z^2(1-α/2) is the value of the standard normal variate corresponding to 1-α/2 for the level of significance,

P denotes the anticipated population proportion,

100(1-α/2)% indicates the confidence level,

d is the absolute precision required on either side of the population estimate.

In this study, the following values for the above parameters have been considered, taking into account the frequency of available cases in the hospital:

(i) Confidence level: 95%

(ii) Anticipated population proportion, P=50%

Since we need to estimate varying proportions, 50% has been assumed, as this proportion yields the highest minimum sample size.

(iii) Absolute precision: d=7%.

For the given input values, the minimum required sample size was calculated to be 196. However, the study includes a sample size of 203, which exceeds the minimum requirement.

Patients with known type 2 DM, aged 18 years and older, either newly diagnosed or on follow-up (diagnosed as per American Diabetes Association 2017 criteria) [21], attending the outpatient department or admitted to wards, were enrolled through a simple random sampling method after we obtained informed consent. A detailed clinical history was collected, and a thorough examination was conducted. Routine investigations were carried out, with special emphasis on complete blood count and renal function tests. Subjects were screened for chronic kidney disease as per Kidney Disease: Improving Global Outcomes (KDIGO) guidelines. Participants with a spot urine albumin-to-creatine ratio >30 mg/gm were considered to have nephropathy and were included in the study [22]. GFR was calculated using the Modification of Diet in Renal Disease (MDRD) formula, and nephropathy staging was conducted according to KDIGO criteria [23]. Patients without nephropathy were excluded from the study. Additionally, patients with a history of chronic diseases such as connective tissue disease, thyroid disorders, malignancies, known platelet disorders, or those on anti-platelet drugs were also excluded. Complete blood count analysis, including haemoglobin, total leucocyte count, total platelet count, and platelet indices like MPV, PWD, PCT and PLCR, was performed using the SYSMEX XN-1000 (Sysmex Corporation, Kobe, Japan) automated haematology analyzer. All clinical and laboratory data were recorded on a predesigned case report form.

Statistical analysis

Data collected during the study were reviewed, codified, and entered into Statistical Package for the Social Sciences (IBM SPSS Statistics for Windows, IBM Corp., Version 24.0, Armonk, NY) for analysis. Age distribution by gender was assessed using the cross-tabulation procedure. Categorical variables like haemoglobin, fasting blood sugar (FBS), post-prandial blood sugar (PPBS), glycated haemoglobin (HbA1C), diabetes duration, hypertension, total platelet count, MPV, PDW, PCT, PLCR, and nephropathy stage were analyzed using the frequency procedure. Parametric tests such as the independent samples t-test were used for normally distributed data, while nonparametric tests were applied to nonnormally distributed data. Comparison of MPV, PDW, PCT, and PLCR with nephropathy stages was analyzed using the nonparametric Kruskal-Wallis test. Statistical significance was defined as p < 0.05.

## Results

A total of 203 patients with type 2 DM with nephropathy were included in the study, with a mean age of 61.7 ± 12.0 years and a male-to-female ratio of 1.5:1. The mean age of male patients was higher than that of female patients (64.4 ± 11.3 years vs 57.5 ± 11.9 years). Table 1 shows the demographic and clinical characteristics of study participants.

**Table 1:** Demographic/clinical characteristics DM: diabetes mellitus; FBS: fasting blood sugar; PPBS: post-prandial blood sugar; HbA1C: glycated haemoglobin in percentage

Demographic/clinical characteristics		N (%)
Gender	Male	122 (60)
	Female	81 (40)
Age	<40 yrs	6 (3)
	40-60 yrs	71 (35)
	>60 yrs	126 (62)
Hypertension	Yes	142 (70)
	No	61 (30)
Duration of DM	<6 years	116 (36.5)
	6-10 years	96 (47)
	>10 years	33 (16.5)
Lab parameters		Mean±SD
FBS in mg/dL		165.2±80.5
2hr PPBS in mg/dL		248.7±93.9
HbA1C		8.8±2.7
Duration of DM in years		8.0±5.2
Hb in gm/dL		10.3±2.0
Total platelet count in lakhs/ μL		236.4±112.6

The platelet indices such as MPV, PDW, PCT, and PLCR were specifically analyzed to assess their correlation with the severity of nephropathy. The mean values of MPV, PDW, PCT, and PLCR in the study population were 11.4 ± 1.7, 15.2 ± 3.8, 0.28 ± 0.11, and 38.9 ± 11.8, respectively. The distribution of these platelet indices across different ranges among the study participants is presented in Table 2.

**Table 2:** Classification of platelet indices among study participants MPV: mean platelet volume; PDW: platelet distribution width; PCT: plateletcrit; PLCR: platelet large cell ratio

Platelet Indices	Range	No.	%
MPV	<7	0	0
	7-9.5	28	13.8
	9.5	175	86.2
	Mean ± SD	11.4 ± 1.7	
PDW	<9	0	0
	9-17	134	66
	>17	69	34
	Mean ± SD	15.2 ± 3.8	
PCT	<0.17	40	19.7
	0.17-0.35	116	57.1
	>0.35	47	23.2
	Mean ± SD	0.28 ± 0.11	
PLCR	<13	0	0
	13-43	130	64
	>43	73	36
	Mean ± SD	38.9 ± 11.8	

Renal function was evaluated using the calculated GFR and KIDGO staging, and it was observed that the majority of patients (63.1%) were in early nephropathy (stages 1­-3), while a smaller percentage of patients (36.9%) were in the advanced stages (stages 4 or 5). The distribution of nephropathy stages among the study participants is shown in Table 3.

**Table 3:** Distribution of cases according to nephropathy stages

Staging	Number of cases	%
Stage 1	45	22.2
Stage 2	41	20.2
Stage 3a	22	10.8
Stage 3b	20	9.9
Stage 4	40	19.7
Stage 5	35	17.2
Total	203	100

All platelet indices were individually compared with the stages of nephropathy in the study participants using the Kruskal-Wallis test, aiming to identify any statistically significant correlation. Table 4 shows the comparison of platelet indices with the nephropathy stage.

**Table 4:** Comparison of platelet indices across nephropathy stages MPV: mean platelet volume; PDW: platelet distribution width; PLCR: platelet large cell ratio; PCT: plateletcrit

Nephropathy Stage	Platelet Indices	Kruskal-Wallis p-value
Stages 1, 2, 3a, 3b, 4, 5	MPV	0.027
Stages 1, 2, 3a, 3b, 4, 5	PDW	0.009
Stages 1, 2, 3a, 3b, 4, 5	PCT	0.001
Stages 1, 2, 3a, 3b, 4, 5	PLCR	0.007

**Figure 1:**
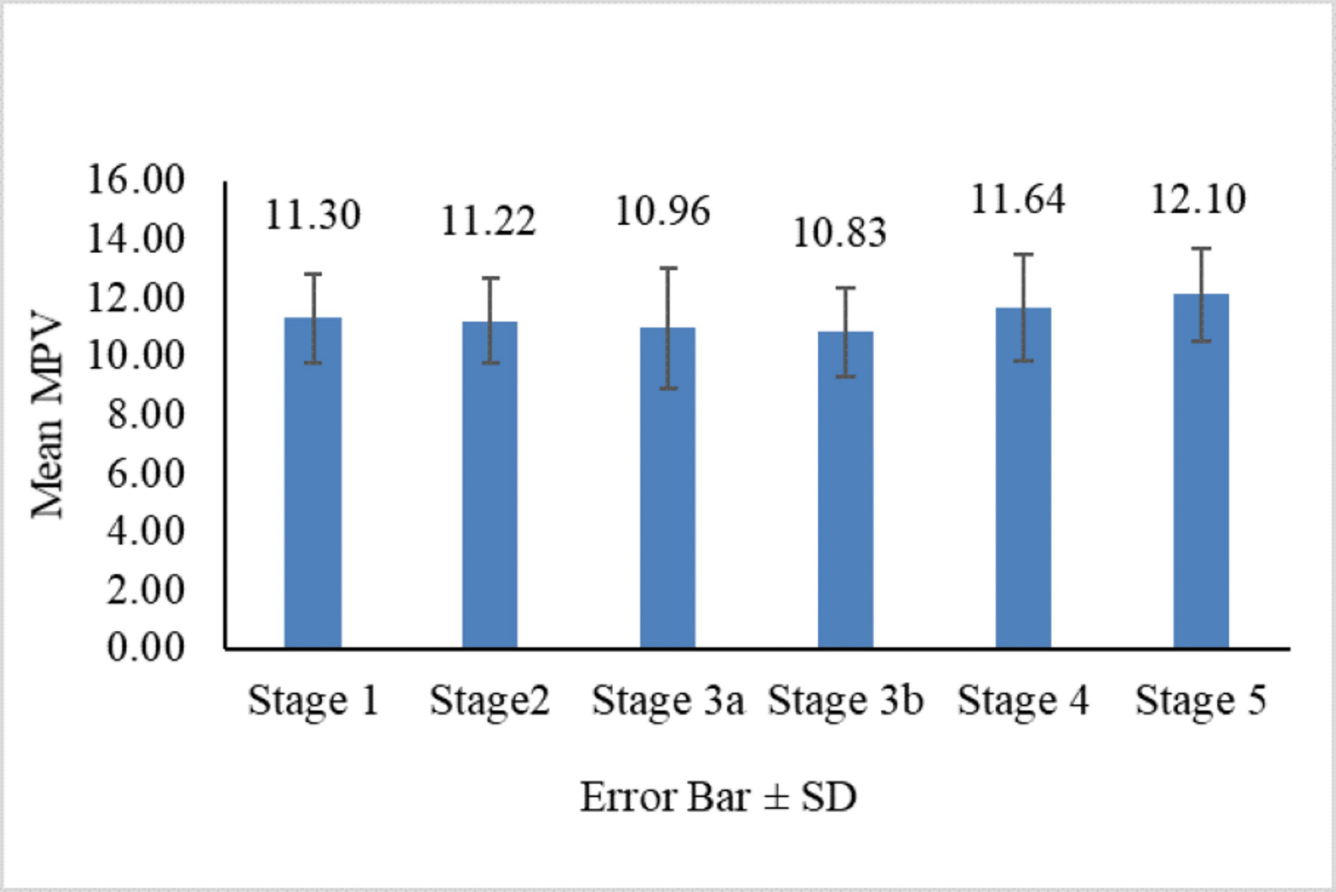
Comparison of MPV across nephropathy stages MPV: mean platelet volume; SD: standard deviation

**Figure 2:**
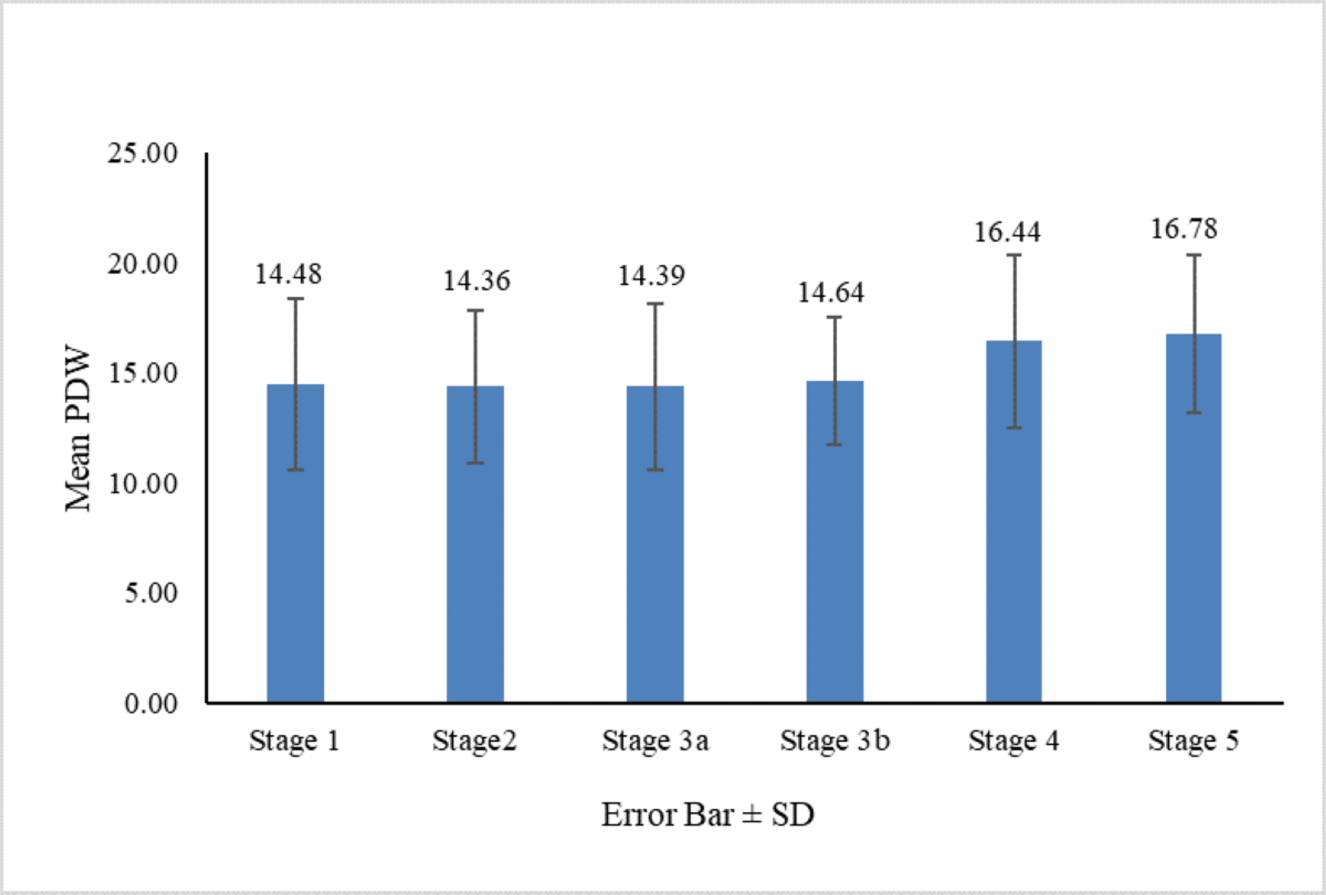
Comparison of PDW across nephropathy stages PDW: platelet distribution width

**Figure 3:**
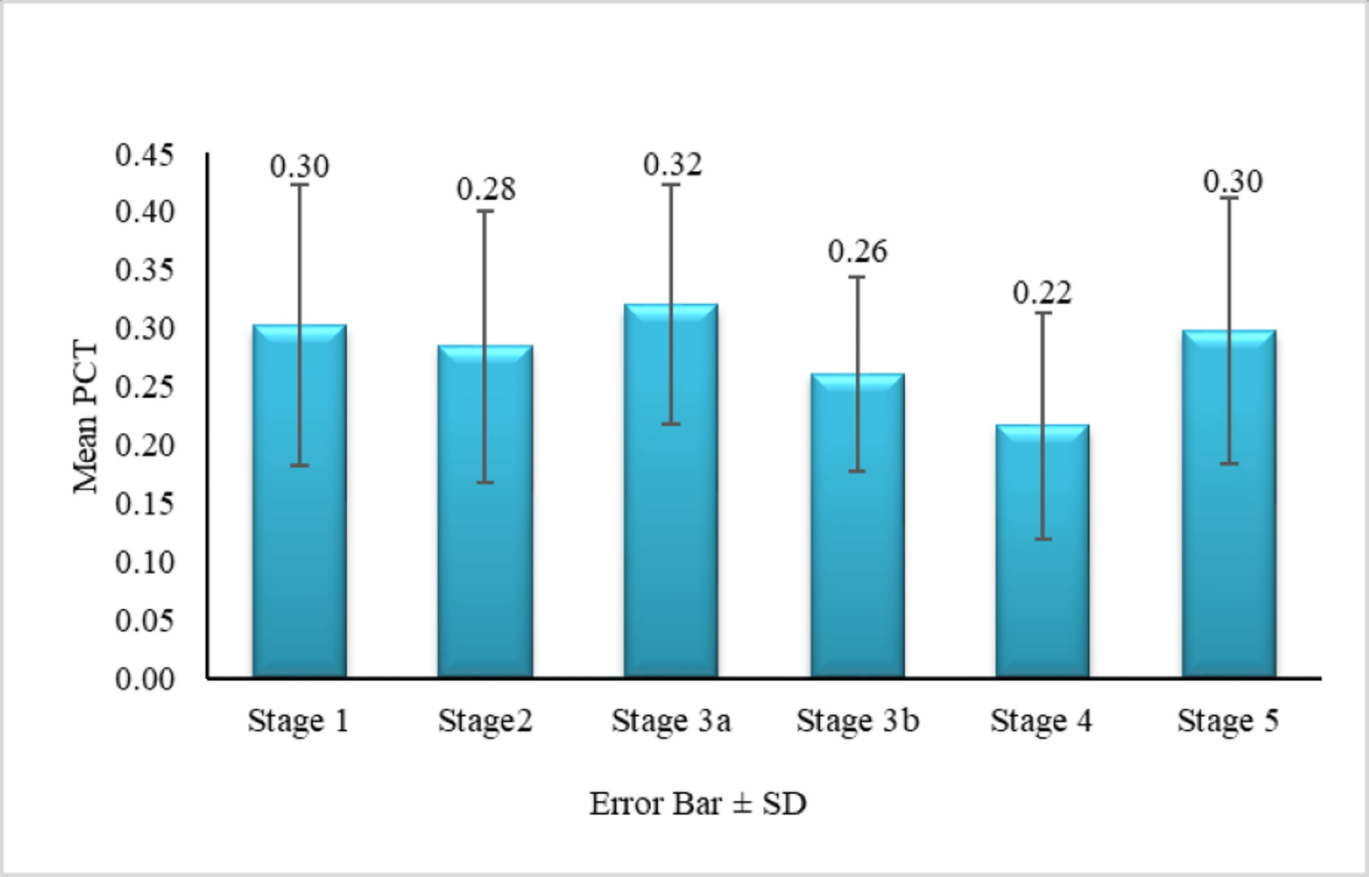
Comparison of PCT across nephropathy stages PCT: plateletcrit

**Figure 4:**
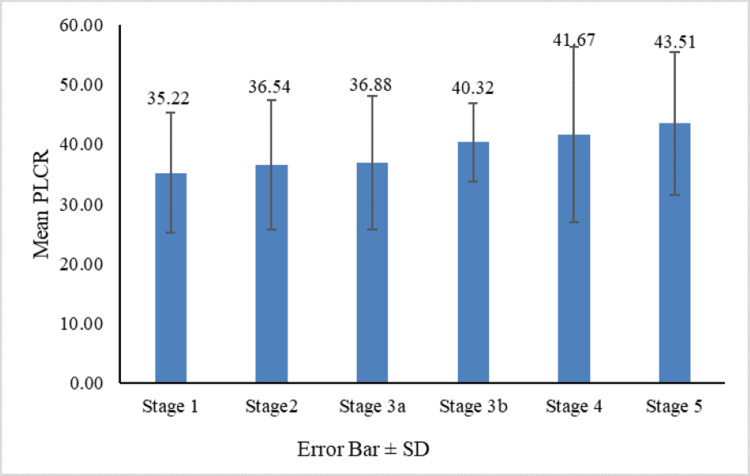
Comparison of PLCR across nephropathy stages PLCR: platelet large cell ratio

Figures 1-4 show the comparison of MPV, PDW, PCT, and PLCR across the nephropathy stage (Kruskal-Wallis test).

Table 5 shows the pairwise comparison of platelet indices (MPV, PDW, PCT, and PLCR) with respect to different stages of nephropathy.

**Table 5:** The pairwise comparison of platelet indices (MPV, PDW, PCT, and PLCR) with respect to different stages of nephropathy MPV: mean platelet volume; PDW: platelet distribution width; PCT: plateletcrit; PLCR: platelet large cell ratio

Mann-Whitney Test	Mean Rank		p-value
MPV and Nephropathy Staging			
Stage 1 vs Stage 5	34.22	48.57	0.006
Stage 2 vs Stage 5	32.61	45.40	0.012
Stage3a vs Stage 5	22.91	32.83	0.028
Stage 3b vs Stage 5	20.48	32.30	0.008
PDW and Nephropathy Staging			
Stage 1 vs Stage 4	36.97	49.79	0.017
Stage 1 vs Stage 5	34.56	48.14	0.009
Stage 2 vs Stage 4	34.56	47.60	0.013
Stage 2 vs Stage 5	32.27	45.80	0.008
Stage 3a vs Stage 5	23.32	32.57	0.040
Stage 3b vs Stage 5	20.68	32.19	0.010
PCT and Nephropathy Staging			
Stage 1 vs Stage 4	52.01	32.86	0.000
Stage 2 vs Stage 4	47.83	34.00	0.008
Stage 3a vs Stage 4	42.73	25.33	0.000
Stage 3b vs Stage 4	36.90	27.30	0.044
Stage 4 vs Stage 5	30.39	46.70	0.001
Comparison of PLCR With Nephropathy Staging			
Stage 1 vs Stage 3b	29.22	41.50	0.016
Stage 1 vs Stage 5	33.02	50.11	0.001
Stage 2 vs Stage 5	31.76	46.40	0.004
Stage 3a vs Stage 5	23.14	32.69	0.034

The platelet indices (MPV, PDW, PCT, and PLCR) were compared across different stages of nephropathy (stages 1, 2, 3a, 3b, 4, and 5) using the Mann-Whitney test. No significant differences were observed in MPV for the following stage comparisons: stage 1 vs. stage 2, stage 1 vs. stage 3a, stage 1 vs. stage 3b, stage 1 vs. stage 4, stage 2 vs. stage 3a, stage 2 vs. stage 3b, stage 2 vs. stage 4, stage 3a vs. stage 3b, stage 3a vs. stage 4, and stage 4 vs. stage 5 (p > 0.05). However, significant differences were observed in the following pairs: stage 1 vs. stage 5, stage 2 vs. stage 5, stage 3a vs. stage 5, stage 3b vs. stage 4, and stage 3b vs. stage 5 (p < 0.05).

The mean rank of PDW did not show any significant differences in the following comparisons: stage 1 vs. stage 2, stage 1 vs. stage 3a, stage 1 vs. stage 3b, stage 2 vs. stage 3a, stage 2 vs. stage 3b, stage 3a vs. stage 3b, stage 3a vs. stage 4, stage 3b vs. stage 4, and stage 4 vs. stage 5 (p > 0.05). However, significant differences in PDW were observed in the following pairs: stage 1 vs. stage 4, stage 1 vs. stage 5, stage 2 vs. stage 4, stage 2 vs. stage 5, stage 3a vs. stage 5, and stage 3b vs. stage 5 (p < 0.05).

The mean rank of PCT did not show any significant differences in the following comparisons: stage 1 vs. stage 2, stage 1 vs. stage 3a, stage 1 vs. stage 3b, stage 1 vs. stage 5, stage 2 vs. stage 3a, stage 2 vs. stage 3b, stage 2 vs. stage 5, stage 3a vs. stage 3b, stage 3a vs. stage 5, and stage 3b vs. stage 5 (p > 0.05). However, significant differences in PCT were observed between stage 1 vs. stage 4, stage 2 vs. stage 4, stage 3a vs. stage 4, stage 3b vs. stage 4, and stage 4 vs. stage 5 (p < 0.05).

The mean rank of PLCR did not show any significant differences in the following comparisons: stage 1 vs. stage 2, stage 1 vs. stage 3a, stage 1 vs. stage 4, stage 2 vs. stage 3a, stage 2 vs. stage 3b, stage 2 vs. stage 4, stage 3a vs. stage 3b, stage 3a vs. stage 4, stage 3b vs. stage 4, stage 3b vs. stage 5, and stage 4 vs. stage 5 (p > 0.05). However, significant differences in PLCR were observed between stage 1 vs. stage 3b, stage 1 vs. stage 5, stage 2 vs. stage 5, and stage 3a vs. stage 5 (p < 0.05).

## Discussion

DM has become a major global health challenge. The rising prevalence of obesity, combined with sedentary lifestyles and increasing stress levels, complicates the prevention and control of hyperglycemia in DM. Hyperglycemia in DM leads to the glycation of various proteins, triggering an inflammatory milieu at the cellular and tissue levels, with increased cytokines levels such as tumour necrosis factor-alpha (TNF-α), interleukin-6, and C-reactive protein [24]. This inflammatory reaction at the tissue level leads to microvascular complications like diabetic nephropathy, neuropathy, and retinopathy. Moreover, metabolic abnormalities in DM activate platelets, which play an important role in both microvascular and macrovascular complications [25,26]. Platelet indices, such as MPV, PDW, PCT, and PLCR, reflect platelet function [27]. Several researchers have established a link between changes in platelet indices and vascular complications in DM, including retinopathy and nephropathy. However, few have explored the association of platelet indices with different stages of nephropathy in DM. In this study, we investigated the correlation between platelet indices and the progression of diabetic nephropathy.

In the present study, 86.2% of subjects had elevated MPV levels, with a mean of 11.4 ± 1.7. This aligns with previous findings by Agrawal BK et al. [28], who reported a mean MPV of 11 ± 2.2, indicating elevated mean MPV levels in diabetic groups compared to non-diabetic controls. Similar trends were observed in studies by Kodiatte TA et al. [29], Zuberi BF et al. [30], and Jindal S et al. [31]. There are several possible explanations for elevated MPV in T2DM. Studies have shown that inflammatory markers are associated with the evolution of overt diabetes, which suggests that DM is an inflammatory condition. MPV has been shown to be associated with a variety of inflammatory conditions in various studies [32]. Also, platelets that have been exposed to inflammatory conditions have shown an increase in size and the number of cytoplasmic granules in response to inflammation. Increased serum levels of inflammatory products in inflammatory diseases may interact with megakaryopoiesis in the bone marrow, resulting in the formation of bigger platelets. In type 2 DM, insulin levels increase in the serum of patients. Animal studies have shown that elevated insulin levels stimulate megakaryocytes to produce larger platelets [33]. Additionally, in the context of hyperglycemia, osmotic swelling may also contribute to the elevated MPV.

In this study, the PDW ranged from 9 to 17 fL, with a mean of 15.2 ± 3.8. This finding is consistent with the results reported by Jabeen F et al. [34] and Dalamaga M et al. [35], who observed mean PDW values of 15.02 and 16.4, respectively, in diabetic groups. These values were significantly higher when compared with non-diabetic control groups. PDW is a direct measure of platelet size and its variability. Elevated PDW indicates the presence of larger, immature circulating platelets, which may result from abnormal platelet activation in long-standing, uncontrolled diabetes, leading to the formation of pseudothrombocytosis [36].

Various researchers have reported differing PLCR values in diabetic patients. In this study, the mean PLCR was 38.9 ± 11.8, significantly higher in diabetic patients. Contrary to this, Khode V et al. [37] and Yilmaz T and Yilmaz A [38] reported significantly lower PLCR levels in diabetic groups compared to non-diabetic controls, with mean PLCR values of 21.33 ± 6.1, 29.4 ± 7.38, and 31.71 ± 2.16, respectively. However, Ashraf S et al. [39] and Sushma KL and Rangaswamy M [40] found significantly higher PLCR levels in diabetics compared to non-diabetics, consistent with the findings of our study. PLCR reflects the proportion of a larger platelet fraction and is associated with platelet volume. An increase in PLCR usually accompanies a rise in newly formed platelets, which are the largest type of platelets. An increase in PLCR may indicate the presence of giant platelets, platelet aggregates, or microerythrocytes. More research is needed to elucidate this marker.

In this study, the mean PCT was 0.28 ± 0.11, which was significantly higher, aligning with the findings of Alhadas KR et al. [41], who also reported higher PCT levels in diabetics. Similarly, Demirtas L et al. [42] found significantly higher PCT levels in type 2 DM patients compared to controls. In contrast, Citirik M et al. [43] found no significant difference in PCT levels between the diabetic group and healthy individuals. PCT is defined as the number of circulating platelets per volume of blood. It is a quantitative test, performed for abnormalities of platelet count, measured as platelet count x MPV/10^7^. In diabetic patients, larger and more reactive platelets contribute to an increased platelet mass, thereby elevating PCT. Although many researchers have investigated the correlation between platelet indices and microvascular complications in diabetes, fewer authors have explored the association between platelet indices and the stages of diabetic nephropathy. In our study, we compared each platelet index with different stages of nephropathy. Among the cases, stages 3b and 3a had the lowest proportions, showing 9.9% and 10.8%, respectively, whereas the highest proportion was observed in stage 1 (22.2%), followed by stages 2, 4, and 5 with 20.2%, 19.7%, and 17.2%, respectively.

When comparing the mean MPV across stages 1-5, stage 5 had the highest mean MPV. There was a significant difference in MPV distribution among nephropathy stages (p = 0.027). Specifically, significant differences in mean MPV (p < 0.05) were observed between stages 1 and 5, 2 and 5, 3a and 5, 3b and 5, and 3b and 4. These results suggest that MPV increases with nephropathy severity. Similarly, the mean PDW was highest in stage 5, with a significant difference in PDW across nephropathy stages (p = 0.009). Significant differences in PDW (p < 0.05) were found between stages 1 and 4, 1 and 5, 2 and 4, 2 and 5, 3a and 5, and 3b and 5. This shows that PDW also increases as nephropathy advances.

Although the mean PCT was highest in stage 3a, the difference in PCT across all stages was statistically significant (p = 0.001). Significant differences in PCT (p < 0.05) were observed between stages 1 and 4, 2 and 4, 3a and 4, 3b and 4, and 4 and 5. PCT levels thus increase with nephropathy severity. In our study, the mean PLCR was highest in stage 5, and the difference in PLCR across stages was statistically significant (p = 0.007). Significant differences in PLCR (P < 0.05) were observed between stages 1 and 3b, 1 and 5, 2 and 5, and 3a and 5. Thus, PLCR also increases with the progression of nephropathy.

Few researchers have examined the correlation between platelet and nephropathy severity. Turgutalp K et al. found higher MPV values in all diabetic patients compared to normal participants (p < 0.05) and reported a positive correlation among MPV, serum creatinine, and proteinuria and a negative correlation with GFR (p < 0.001 for all; r values: 0.72, 0.82, and -0.92, respectively) [44]. Coskun YE et al. also observed a positive correlation between CKD stage and MPV in diabetic male patients [45]. In a retrospective study by Ju HY et al., MPV was negatively associated with estimated GFR in CKD patients, with MPV increasing in parallel with CKD progression [46]. Tamandon MR et al. observed that changes in MPV had an inverse relationship with creatinine levels in hypertensive patients (r = -0.484, p = 0.049), concluding that MPV is not a reliable prognostic marker for kidney disease progression [47].

However, Aashitha PM et al. reported that MPV, PDW, and PCT did not correlate with CKD stages, but these indices were significantly higher in stage 5 compared to other stages [20]. Many researchers focus solely on MPV, except Aashitha PM et al., who evaluated PDW and PCT. In our study, we assessed four platelet indices and found a positive correlation among MPV, PDW, PLCR, and PCT.

## Conclusions

Platelet indices are a simple and cost-effective method for detecting microvascular complications in type 2 DM. Platelet indices may serve as a reliable marker of diabetic nephropathy, as supported by both this and other studies. Additionally, platelet indices can be considered surrogate markers and predictors of nephropathy severity in type 2 DM patients. However, due to the limited number of studies in this area, further research is required to validate the utility of platelet indices.
